# Voluntary ‘donations’ versus reward-oriented ‘contributions’: two experiments on framing in funding mechanisms

**DOI:** 10.1007/s10683-022-09759-6

**Published:** 2022-07-25

**Authors:** Maja Adena, Steffen Huck

**Affiliations:** 1 WZB Berlin, Reichpietschufer 50, D-10785 Berlin, Germany; 2 UCL London, Department of Economics, Gower St, WC1E 6BT, London, England

**Keywords:** Crowdfunding, Field experiment, Framing, C93, D64, D12

## Abstract

In an artefactual field experiment, we implemented a crowdfunding campaign for an institute’s summer party and compared donation and contribution framings. We found that the use of the word ‘donation’ generated higher revenue than the use of ‘contribution.’ While the individuals receiving the donation framing gave substantially larger amounts, those receiving the contribution framing responded more strongly to reward thresholds and suggestions. An additional survey experiment on MTurk indicated that the term ‘donation’ triggers more positive emotional responses and that emotions are highly correlated with giving. It appears that making a donation is perceived as a more voluntary act and is thus more successful at generating warm glow than making a contribution. We surmise that this extends to other funding mechanisms.

**Supplementary information:**

The online version contains supplementary material available at 10.1007/s10683-022-09759-6.

## Introduction

Charitable giving, public good provision, and crowdfunding all have one thing in common: agents give money to finance a nonprivate good. The main difference between the three lies in the nature of the good for which money is being collected. While the beneficiaries of charitable giving are typically *other* people and the beneficiaries of public goods are by definition everyone, the beneficiaries of many crowdfunding campaigns often include the contributors. Neither charitable giving nor public good provision mechanisms typically involve rewards for donors; by contrast, crowdfunding campaigns often involve nested reward schemes for different contributions.^1^

Regardless of the nature of the beneficiaries of a funding mechanism, the question arises as to how to describe to potential contributors the act of giving money. In public good games, it is common to refer to the money that is given as a ‘contribution,’ while in charitable giving settings, money given is mostly called a ‘donation.’ In this paper we explore whether this choice of wording matters for behaviour. While we do this in the context of a crowdfunding campaign,^2^ we believe that our results also have implications for other funding mechanisms.

Specifically, we implemented a crowdfunding campaign to finance one occurrence of an institute’s annual summer party. In previous years, a ‘donation box’ had been displayed in a prominent location during the party, which frequently led to shortfalls in financing. This time, a crowdfunding campaign was announced around 20 days in advance via personalized e-mails. The campaign offered a multitude of incentives to increase giving. Those incentives were available to all e-mail recipients alike and included rewards like vouchers for tournaments and games and matching for early gifts. Three e-mail reminders were sent. Our setting was a relatively small community consisting of an institute’s more than 500 affiliates and friends; expected attendance at the party was between 150 and 200 guests.^3^

We implemented a subtle treatment manipulation in the wording of our e-mails, that is, we referred to either donations or contributions. In order to learn more about the mechanism driving giving behaviour in both conditions, we also varied non-binding suggestions, which were either €10 or €20. This was the second dimension of our 2 × 2 design. Additionally, we studied the responsiveness to other incentives offered (without experimental variation) depending on the frame. Specifically, we analysed gift levels relative to the reward thresholds and self-selection with respect to the timing of gifts. Regarding timing, early gifts were matched with a fixed amount offered by an anonymous sponsor, but later gifts were not matched.^4^

While the term ‘donation’ has a clear meaning linked to charitable giving, ‘contribution’ has multiple meanings. Some of these meanings relate to charitable giving, but others are more related to duties. In Fig. 4 in the Appendix we present word association maps that show different meanings and their connections. They suggest that the act of donating is more self-oriented, while contributing invokes a notion of joint participation. On Google Trends, search terms combined with the word ‘donation’ mostly relate to charitable giving (blood, organ, plasma, Goodwill, Salvation Army, Red Cross, clothing), while those combined with ‘contribution’ mostly relate to individual accounts, savings, or insurance and ask questions about their regulation (see the lists in Table 9 in the Appendix). The search term ‘donation’ is approximately 20% more common than ‘contribution,’ and ‘charitable donation’ occurs 71% more often than ‘charitable contribution’ in Google searches.^5^ While charities predominantly use the term ‘donation,’ they do employ the term ‘contribution’ in some instances (see Table 10 in the Appendix).

Relatedly, in the literature on charitable giving, Andreoni (1995) documented that framing the same task as implying a positive externality rather than a negative externality generates more giving. This result has been replicated several times by, among others, Sonnemans et al. (1998) and Park (2000). This line of research concludes that positive frames are more successful at stimulating warm glow than negative frames.^6^

In line with the above-mentioned research, we expected that the more unique meaning of the term ‘donation’ and its connotation with voluntary charitable giving would increase giving by intensifying warm glow when compared to the term ‘contribution.’ Given the different connotations, we also expected that gifts in the contribution frame would be more responsive to suggestions and rewards than those in the donation frame.

In order to further investigate the reasons for our treatment effects, we conducted an additional survey experiment on Amazon Mechanical Turk (MTurk), where we measured emotional responses to the two frames. In line with our conjecture, we found more positive emotional responses to the donation frame and also show that emotional responses are correlated with behaviour in a public good game—highlighting that the main result from our field experiment extends beyond the crowdfunding setting.

Our paper contributes to three strands of literature: the large literature on framing effects (including the papers cited above), the literature on emotions and economic decision-making, and the nascent literature on crowdfunding that emerged in the 2010s. Mollick (2014) provides an early descriptive study of almost 50,000 crowdfunding projects, and Agrawal et al. (2014) provide an early overview of the basic economic principles governing the crowdfunding market. Strausz (2017) contributes a formal economic model of crowdfunding highlighting the tension between its screening function in the presence of demand uncertainty and moral hazard. Belleflamme et al. (2015) studied the economics of crowdfunding platforms and illustrate how externalities between crowdfunding projects lead to the natural emergence of platforms.

While most existing empirical studies of crowdfunding make use of observational data (for example, Meer, 2014, Argo et al., 2020), there are also a small number of experiments on crowdfunding. Cason and Zubrickas (2017, 2020) and Cason, Tabarrok, and Zubrickas (2020) conducted laboratory experiments in which they explored different incentive schemes such as bonuses for early contributions. Similarly, in a web-based experiment, Ansink et al. (2017) tested the effects of seed money and the impact of the attraction effect. In a field experiment, Burtch et al. (2015) studied the effects of privacy. Castillo et al. (2017) show how including a feature to ask friends on Facebook for additional donations increased giving on a crowdfunding platform. Our study is the first to document a substantial framing effect in crowdfunding campaigns.

In addition to the literature on framing, the literature on nudging often investigates the effects of small changes in the design of choice architecture or in the precise choice of wording. A recent meta study of the effects of nudging in the context of tax collection can be found in Antinyan and Asatryan (2020). They show that emphasizing deterrence is more effective than emphasizing tax morale. In contrast to standard nudging interventions, we should also note that our variation is extremely minimal, akin to a one-word nudge. In addition, the terms that we used—‘donation’ and ‘contribution’—have a similar meaning, do not change the information structure, and do not affect the choice architecture.

As the effect that we document appears to be mediated through associated emotions, our paper also makes a contribution to the literature on emotions and economic decision-making. The role of emotions for contribution games was documented earlier by Drouvelis and Grosskopf (2016), who show that anger reduces contributions in a public good game with punishments. While they manipulated emotions before the actual decision through video clips screened to subjects,^7^ the emotional response in our subjects was triggered simply by different wordings in the instructions and measured in our online survey experiment. Emotions have also been shown to be relevant in other contribution contexts, such as tax compliance (Enachescu et al., 2019) and pro-environmental donations (Ibanez, Moureau, and Roussel, 2017), with positive emotions being associated with better compliance.^8^

In light of these studies, it is perhaps not very surprising that emotions also matter for contributions in a crowdfunding campaign. What appears non-trivial is that different emotions can be triggered through the slightest change in wording.

The remainder of the paper proceeds as follows. In Sect. 2 we present the basic idea and our hypotheses. In Sect. 3 we describe the design and implementation of the crowdfunding campaign, followed by the results from the field experiment in Sect. 4. Sect. 5 presents the additional experiment on MTurk measuring emotional responses, and Sect. 6 concludes.

## The basic idea and hypotheses

We designed a crowdfunding campaign with three basic characteristics. Individuals (i) were asked to make a contribution to a nonprivate good, (ii) received an implicit suggestion for an amount that they might have deemed appropriate, and (iii) were offered staggered rewards for contributions that met certain thresholds. We believe that this captures some of the most common features of crowdfunding campaigns. Our main treatment variation was the wording we used for the contribution as such. In one treatment, the contribution was referred to as a contribution; in the other, a donation.

Based on the aforementioned word associations maps and most common Google search phrases, we suspected that the term ‘donation’ is associated with the positive sentiments of voluntary action and charity, while the term ‘contribution’ is more associated with the negative sentiments of duty and taxation. From this, we derived the following three hypotheses for the crowdfunding campaign:

### Hypothesis 1

*The donation frame will lead to higher gift levels than the contribution frame.*

The logic behind this hypothesis is simple. If the term ‘donation’ were to trigger a more positive emotional response, we should, in line with previous findings, expect more generosity.

### Hypothesis 2

*The donation frame will lead to a higher share of individuals choosing gift levels over and above the different reward thresholds.*

The logic for the second hypothesis is derived from the idea that the term ‘donation’ is associated with an element of charity and that charity as such cannot be signalled to others or to oneself when the amount given appears to be driven by a reward.

### Hypothesis 3

*Individuals in the contribution frame will be more responsive to suggestions: the distance between chosen gifts and suggested amounts will be smaller in the contribution than in the donation frame.*

The logic for the third hypothesis stems from the observation that the term ‘contribution’ is associated with a notion of duty and that duties can be fulfilled by following (implicit) suggestions.

## Design and implementation of the crowdfunding campaign

Each year one of the departments of the research institute is responsible for the organization of a summer party. The fields represented at the institute include sociology, political science, law, and economics. In 2016, the department of economics was responsible for the organization and financing of the summer party.^9^ As usual, almost 550 employees, guests, and affiliated researchers were invited. Around half were employed as researchers (including PhD candidates and student research assistants), one quarter worked in administration, and the final quarter was made up of guests, affiliated researchers, alumni, and friends. The party usually involves free drinks and a barbecue or alternatives financed through monetary contributions, a salad and cake buffet organized through in-kind contributions, live music, and an entertainment program with games and humorous speeches.

Instead of employing a donation box, which in previous years had led to shortfalls in financing, this time the invitation e-mail announced a crowdfunding campaign to take place before the summer party. More specifically, there were four different versions of e-mails sent out 20 days before the party. A 2 × 2 design involved one treatment pair with a variation in wording and one pair with two different suggestions regarding the gift amounts. The e-mail recipients were asked to either contribute or donate money and/or make a pledge to a potluck buffet of salads and cakes (a buffet pledge).^10^ In addition, suggestions were introduced in the first e-mail with the following sentence: ‘If the average monetary donation (contribution) is €20 **<**€10**>**, we need 100 < 200 > participants in the campaign to cover the expected costs.’ The same sentence was repeated in the final reminder e-mail. This formulation mirrors the variations in Adena, Huck, and Rasul (2014). The total amount collected to date was posted and updated once a day on the institute’s intranet as well as communicated via reminder e-mails over the course of the campaign.

We also implemented some additional incentives that were equal for all versions of e-mails and aimed at making participation in the campaign more attractive. First, we offered various nested rewards by levels of gifts, with thresholds at €5, €10, €20, €30, and €100. The rewards included vouchers for participation in tournaments and games, and a rare book for the highest gifts. A buffet pledge was valued at €10, the average price that the organizers would have had to pay to a professional caterer for a cake or salad, and added to the monetary gift when determining the reward. Second, we offered a fixed match of €5 by an anonymous sponsor for early gifts; this was not counted towards the reward. In addition, it was announced that any surplus money would be donated to a refugee project (see Appendix D for details of the mailing). In addition to the first e-mail, three reminders were sent. The e-mails were sent in English,^11^ since a large proportion of the institute’s staff is international and has little or no command of the local language.

In the donation treatment, the word ‘donation’ appeared 19 times in the first e-mail, once in the first (short) reminder, twice in the second reminder, and four times in the third reminder, whereas the word ‘contribution’ was never used. Each time the e-mail was sent, all previous e-mail communications were appended such that with the third reminder the total word count of ‘donation’ was 26. The contribution treatment involved the same number of instances of the word ‘contribution’ and no use of the word ‘donation.’

We implemented block (strata) randomization based on the available individual characteristics, which in turn were based on membership in email lists such as ‘female,’ ‘postdocs,’ ‘PhD students,’ and those for different departments or different administrative divisions, amongst others.^12^ More specifically, we sorted the data according to the following dummy variables and in the following order: professor, female, data management unit, press and communication unit, doctoral students, postdocs, units IV, I, II, III, V, administration, secretaries, IT unit, student research assistants, and library. Next, in each consecutive group of four individuals (our blocks), we assigned one of the four experimental treatments at random.^13^ We applied the block randomization in order to increase balance and subsequently precision. All variables used for the randomization and mean comparisons between different treatments can be seen in Table 7 in the Appendix. The given sample size of 545 individuals allows us to detect a standardized effect size (Cohen’s d) of 0.24 with alpha equal to 0.05 and power equal to 0.8 in a simple randomized experiment. By applying block randomization, we additionally increased power and therefore efficiency by reducing the residual variance.^14^

By choosing personalized e-mails, we aimed to reduce spillovers between treatments. We cannot rule out that recipients discussed the party with one another. But since the differences between e-mails were rather subtle, they likely went unnoticed, and no one mentioned to us that they had become aware of the variation. If there was some awareness about treatment differences, for which we do not have any evidence, our results would constitute the lower bound of the true treatment effects.^15^

Before proceeding to our results in the next section, we want to briefly reflect on how our setup compares to other funding mechanisms. In Table 1 we compare typical crowdfunding, typical public good games, and typical fundraising environments to our own experiment. While our setup does tick all the boxes for crowdfunding, it is closely related to both fundraising and public good games such that we would expect our results to also speak to other realms.

**Table 1:** Differences between crowdfunding, public goods, fundraising, and this experiment

	Public goods	Fundraising	Crowdfunding	This experiment
Beneficiaries	Everyone	Other people (everyone for certain charitable goals)	Contributors (other people in donation-based form)	Contributors (everyone at the institute)
Goods or services in return for payment	No	Typically no, but can include a lottery or small gifts	Typically yes, rewards possible	Yes, rewards included
Threshold	Typically no, but can be spelled out	Typically no, but can be spelled out	Typically yes, usually provided but not always binding (for example, JustGiving, betterplace.org)	Yes, implicitly spelled out; not binding but effectively affecting the amount of good provided
Visibility of amounts collected so far	Typically no, but can be spelled out	Typically no, but can be spelled out	Yes, usually provided	Yes, provided in reminder e-mails and updated once a day on the intranet

## Results

The campaign achieved a total of 130 gifts (monetary, buffet, or both),^16^ which is close to the expected participation at the party of around 150 to 200, including family members. Relative to the number of e-mails sent, the response rate was 24%. The average monetary gift was €12 and the median €10. Fig. 2 in the Appendix presents the number of gifts by day and suggests the importance of reminders, since most gifts came in shortly after the reminders had been sent out. Most gifts were exactly equal to the amounts specified in the reward scheme (€5, €10, €20, €30, €100), but there were also a few other amounts. There were eight donations larger than €20, including two €100 donations. Overall, the campaign was successful in collecting enough money to cover the costs of the event and even surpassed the announced monetary threshold of €2,000 when everything is included: the final sum of €2,241 comprises €1,506 in monetary gifts, 34 buffet pledges valued at €340, and an additional €395 from the matching scheme. After all costs had been covered, the surplus of €275 was donated to a refugee program in line with the announcement in the e-mails.

Table 2 summarizes the outcomes by treatments alongside simple comparisons by the mean of a t-test or a test of proportions. The use of the word ‘donation’ rather than ‘contribution’ resulted in a slightly higher response rate (non-significant), much higher average positive monetary gifts (borderline significant at p < 0.1), and a much higher overall monetary return (significant at p < 0.05). The effects are very similar once the buffet pledges are included. In Table 3, Column I, we test Hypothesis 1 in an OLS regression. We regressed unconditional amounts given on the donation treatment dummy, controlling for block fixed effects and basic characteristics.^17^ Panel A accounts for monetary gifts only, while Panel B includes buffet pledges monetized at a value of €10 each. In line with Hypothesis 1, we find higher revenue in the donation frame. The difference is significant at p < 0.5, and the increase in giving is as large as 80% from the average in the contribution frame.

**Table 2:** Results of different wording

Treatment	Contribution		Donation		T-test p-value	Test of proportions p-value
Panel A: only monetary gifts						
Number of subjects	273		272			
Number of monetary gifts	56		64			
Share of monetary gift	0.205	(0.024)	0.235	(0.026)		0.3955
Monetary return per mail in €	1.963	(0.279)	3.327	(0.634)	0.049	
Average positive monetary gift in €	9.571	(0.744)	14.141	(2.218)	0.067	
Minimum in €	5		5			
Median in €	10		10			
Maximum in €	30		100			
Share of gifts €5–6 conditional on giving	0.429	(0.066)	0.406	(0.061)		0.805
Share of gifts €10 conditional on giving	0.411	(0.066)	0.297	(0.057)		0.192
Share of gifts €15 and more conditional on giving	0.161	(0.049)	0.297	(0.057)		0.079
Panel B: including buffet pledges monetized at €10						
Number of buffet gifts	16		18			
Share of buffet gifts	0.059	(0.014)	0.066	(0.015)		0.7357
Total number of gift givers	61		69			
Overall response rate	0.223	(0.025)	0.254	(0.026)		0.3958
Return in € per mail including buffet pledges monetized at €10	2.549	(0.345)	3.989	(0.659)	0.053	
Average positive gift in € including buffet pledges monetized at 10€	11.410	(0.858)	15.725	(2.026)	0.063	

Note: Standard errors in parentheses; two-sided tests

**Table 3:** Treatment effect on revenue and accounting for potential outliers

Winsorizing level:	No winsorizing	€90	€80	€70	€60	€50	€40	€30	drop €100	drop €50+
	I	II	III	IV	V	VI	VII	VIII	IX	X
Panel A: only monetary gifts										
‘Donation’	1.553^**^ (0.636)	1.478^**^ (0.602)	1.402^**^ (0.570)	1.326^**^ (0.541)	1.251^**^ (0.515)	1.175^**^ (0.492)	1.064^**^ (0.463)	0.952^**^ (0.440)	0.879^*^ (0.448)	0.705^*^ (0.419)
Observations	544	544	544	544	544	544	544	544	542	541
*R* ^2^	0.409	0.408	0.406	0.403	0.399	0.394	0.390	0.384	0.363	0.370
Panel B: including buffet pledges monetized at €10										
‘Donation’	1.625^**^ (0.690)	1.550^**^ (0.659)	1.474^**^ (0.630)	1.399^**^ (0.604)	1.323^**^ (0.580)	1.247^**^ (0.560)	1.136^**^ (0.536)	1.062^**^ (0.507)	0.951^*^ (0.523)	0.788 (0.503)
Observations	544	544	544	544	544	544	544	544	542	541
*R* ^2^	0.398	0.396	0.393	0.390	0.386	0.382	0.377	0.374	0.359	0.357

Note: OLS regressions. The outcome variables are unconditional gifts excluding (Panel A) or including (Panel B) buffet pledges monetized at €10. Robust standard errors in parentheses. Controls include block fixed effects and dummies for female, data management unit, press and communication unit, doctoral students, postdocs, units I, II, III, IV, administration, IT unit, student research assistants, and library. ^*^p < 0.10, ^**^p < 0.05, ^***^p < 0.01

**Table 4:** Distribution of gift values

Gift value in €	0	5	6	10	15	20	25	30	35	40	50	100	Total
Panel A: only monetary gifts													
‘Contribution’	217	23	1	23	1	7	0	1	0	0	0	0	273
‘Donation’	208	26	0	19	5	7	1	2	1	0	1	2	272
Total	425	49	1	42	6	14	1	3	1	0	1	2	545
Panel B: including buffet pledges monetized at €10													
‘Contribution’	212	19	1	22	5	13	0	0	0	1	0	0	273
‘Donation’	203	19	0	18	12	13	1	2	1	0	1	2	272
Total	415	38	1	40	17	26	1	2	1	1	1	2	545

Note: Gift thresholds that resulted in a reward are underlined

One might be concerned that the effect was driven by outliers, since the maximum monetary donation in the donation frame was €100 compared with €30 in the contribution frame (€40 when we include buffet pledges monetized at €10). For this reason, in Table 3, Columns II-VII, we repeated the specification from Column I and apply, step-by-step, declining caps on donation amounts. While the raw maximum donations are equal to €100 (Column I), each next column winsorizes donations at the specified lower value up to €30. We see that while the estimate of the treatment effect declines (as the average donation and standard deviation do) over decreasing caps, the coefficients remain significant at p < 0.05. In the last two columns, we repeated the above specification without a cap but removed the large gifts. In Column IX we removed the two gifts of €100 (both in the donation frame), and in Column X we removed gifts of €50 or more (three in the donation frame). The treatment effect is still positive and significant at p < 0.1 except for the last cell. Overall, we conclude that the treatment effect was not driven by outliers. In Fig. 5A in the Appendix we include a further robustness check based on the coefficients from Table 3, Column I: a randomization inference test that has become common recently (Heß, 2017; Young, 2018; Cohen and Dupas, 2010). Fisherian randomization inference provides the means to assess whether an observed realization could be observed by chance even if the treatment were to have had no effect. This test permutates the treatment and control status in the sample and reestimates the coefficients using this placebo assignment multiple times (we set this to 5,000). The results show that it is unlikely that the results that we observe arose by chance.

Table 4 shows the numbers of gifts of different monetary values (Panel A) and gifts including buffet pledges monetized at €10 (Panel B) in the two frames. First, there are more gifts in higher categories in the donation frame. There are seven gifts valued at €25 or more in the donation frame compared to only one in the contribution frame, and 19 (32 in Panel B) gifts valued at €15 or more in the donation frame compared to nine (19 in Panel B) in the contribution frame. The share of gifts valued at €15 or more is significantly higher in the donation frame (see bottom rows of Panel A in Table 2).

Second, there are more gifts in the donation frame that do not correspond to a threshold value for a reward. More specifically, in Table 4, Panel B, there are 15 such gifts in the donation frame and only seven in the contribution frame. Glazer and Konrad (1996) present evidence on bunching donations at the bottom of different published categories. For example, they report that 68% of gifts made in the range of US$1,000–4,999 at Carnegie Mellon University were exactly $1,000. While 68% might appear large, 32% chose to give more than required in order to be listed as donors in that particular category. In a similar vein, Birke (2020) documents in an MTurk experiment that a substantial fraction of subjects performed more voluntary tasks for a charity than necessary for a performance bonus. Moreover, more subjects performed two or more tasks above the bonus level if their behaviour was being observed by others. Birke explains that subjects signal their prosociality by separating from bonus-motivated types. As the amount above the reward level was not observed by others in our case, we think that the choice of higher levels is linked to self-signalling and that the difference between the donation and contribution frame is due to the voluntary component of a donation frame, which is weakened in the contribution frame. If a contribution is perceived as an obligation, then there is no point in signalling prosociality. Altogether, we confirm Hypothesis 2.

Next, we look at the distance between the value of gifts and the suggested amounts in more detail.^18^ Table 5 shows that the distance to the suggested amount is almost 40% larger in the donation frame. There is also more variance in gift amounts in general in the donation than in the contribution frame (Columns III and VI, significant difference according to the variance-comparison test). These results are in line with Hypothesis 3.

**Table 5:** Distance to suggested amounts and variance

	Only monetary gifts			Including buffet pledges monetized at €10		
Treatment	Number of subjects	Distance to the suggested amount	Standard deviations from the mean	Number of subjects	Distance to the suggested amount	Standard deviations from the mean
	I	II	III	IV	V	VI
‘Contribution’	56	7.393	9.571	61	6.787	11.410
		(0. 683)	(0.744)		(0.737)	(0.858)
‘Donation’	64	10.234	14.140	69	9.493	15.725
		(1.858)	(2.218)		(1.734)	(2.026)
One-sided t-test p-value		0.086			0.086	
Variance-comparison test p-value			0.000			0.000
Variance-comparison robust test p-value			0.004			0.037

Finally, we comment on behaviour concerning the match (see also Table 8 in the Appendix). A match of €5 by an anonymous donor was offered for all gifts made before a prespecified deadline. Although the match increased the gift received, it was not counted against the reward that donors received from contributing a certain amount. Therefore, individuals who wanted to increase the total amount collected should have chosen to give early, while those who were only interested in rewards might have given equally later. We also expected out-of-pocket gifts with a match to be lower, following the literature about the crowding-out effect of third-party transfers on charitable giving (see, for example, Huck and Rasul, 2011; Huck, Rasul, and Shephard, 2015; Adena and Huck, 2017). While the number of late gifts without the match was equal in both frames, there were 43 early gifts in the donation frame compared to 36 in the contribution frame.^19^ The level of monetary gifts was in both frames lower with the match. Overall, it appears that the match was more successful at stimulating additional gifts in the donation frame.

## An additional experiment on MTurk measuring emotional responses

In order to parse out the mechanism behind the differences in behaviour in our two different frames, we conducted an additional survey experiment with 985 participants on the MTurk platform.^20^ Subjects were placed in an artefactual situation in which they were asked, depending on the treatment, to ‘donate’ or to ‘contribute’ to a public good. We subsequently measured their feelings using the Geneva Emotional Wheel (GEW).^21^ The GEW measures 20 different emotions that are organized on a circle. The two main dimensions of the circle reflect the extent to which emotions are aligned with feelings of being in control (the vertical axis) and the positivity or negativity of emotions (the horizontal axis).

We implemented one-shot public good games with staggered rewards at a number of thresholds as in our summer party crowdfunding campaign. Subjects played in groups of five, and each subject had an endowment of US$2 from which they could choose how much to donate or contribute to a group account. Payments into the group reaching a threshold of $5 were doubled and shared equally among all subjects; payments below that threshold were not doubled but still shared equally. Payments that exceeded certain thresholds were met with a symbolic reward and an individual rebate. Specifically, at $0.20 subjects received a downloadable certificate called the ‘bronze contributor/donor recognition award’; at $0.40 they received a ‘silver award,’ at $0.80 a ‘gold award,’ and at $1.60 a ‘platinum award.’ Additionally, they were offered a rebate of $0.05 for payments above $0.40, $0.10 for payments above $0.80, and $0.20 for payments above $1.60. Notice that none of these rebates affects the equilibrium prediction of zero payments for selfish rational agents. Each subject received a baseline payment of $0.50, independent of the game outcome. After their choice, subjects were asked to quantify how strongly they experienced the 20 different emotions that feed into the GEW (see Appendix E for detailed instructions).

Average payments into the group account were close to $1.10 under both frames, with almost identically appearing distributions and no treatment effect, as documented in Table 6, Column I. There is however a difference in groups’ abilities to meet the $5 threshold that triggered group payments to be doubled. Under the donation frame, 82.28% of groups reached that threshold, compared to 74.17% under the contribution frame, with higher resulting payouts for the donation frame. While those differences are not statistically significant at conventional levels, we did find a significant treatment effect when examining emotions, and once we explore how emotions map onto payments we will see why there were no effects on choices in the MTurk setting.

**Fig. 1:**
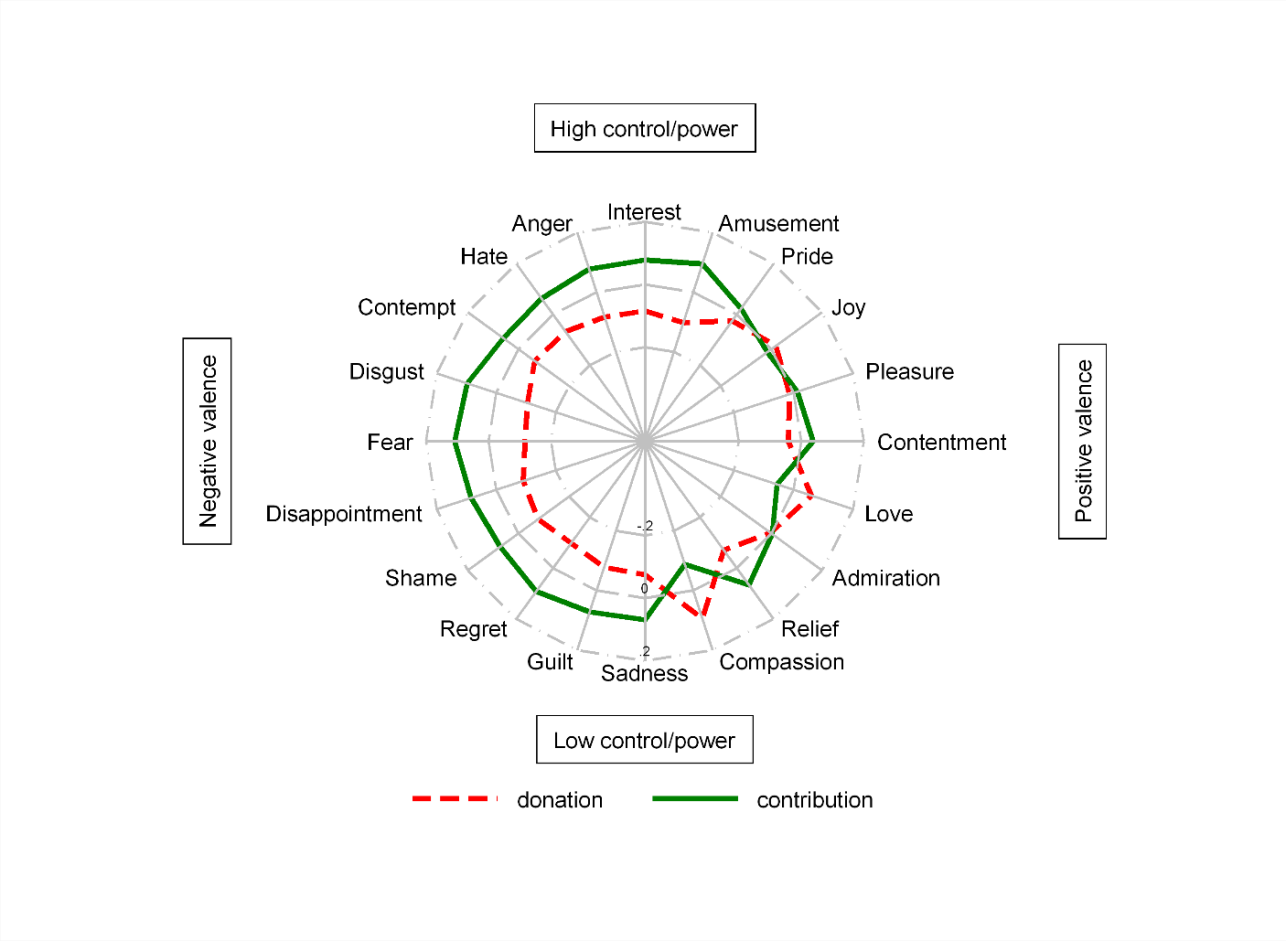
‘Donations’ versus ‘contributions’ on the GEW. (Note: All emotion variables are standardized with mean zero and standard deviation equal to one. The dashed line presents the deviation of the mean in the donation treatment from the overall mean (in terms of standard deviations). The solid line presents the deviation of the mean in the contribution treatment from the overall mean)

Our measurements of emotional responses are presented in condensed form in Fig. 1 and in more detail in Table 11 in the Appendix. In Fig. 1, which shows the GEW, all emotion variables are standardized with mean zero and standard deviation equal to one, chosen because of stark differences on the scale between different emotions. It is easy to see that the contribution frame is associated with more negative feelings than the donation frame: the two frames are roughly two standard deviations apart across the entire left side of the GEW. In terms of positive emotions, the two frames generated much more similar responses, though donations are associated with stronger feelings of ‘love’ and ‘compassion.’

These results appear to be in line with what word maps and Google Trends had suggested: as the term ‘contribution’ implies far less voluntary sentiment and is more reflective of an obligation, it also evokes more negative emotional responses.

In a second step, we completed two regression exercises. In Table 6, Column II, we regress a simple index capturing negative emotions (the sum of negative emotional responses, standardized) on the treatment, while in Column III we regress payments into the group account (standardized) on the negative emotion index and a treatment dummy. An interesting pattern emerges. As is to be expected from inspecting Fig. 1, we found a strong treatment effect on the negative emotion index. We also found that negative emotions significantly reduced contributions, in line with the findings of Drouvelis and Grosskopf (2016), discussed above. However, this relationship is significantly attenuated under the contribution frame. It appears that the subjects in our MTurk treatment tried to keep their negative emotions in check more so when we shocked them upwards in our contribution frame. It is this attenuation that leads to the absence of a treatment effect on average payments into the group account in this particular setting.

**Table 6:** Results of the MTurk experiment

Outcome:	Gifts, standardized	Sum of negative emotions, standardized	Gifts, standardized
	I	II	III
‘Contribution’	0.008 (0.064)	0.207^***^ (0.063)	0.051 (0.063)
Sum of negative emotions, standardized			-0.294^***^ (0.049)
‘Contribution’ x sum of negative emotions, standardized			0.190^***^ (0.065)
Observations	985	985	985
*R* ^2^	0.000	0.011	0.036

Note: Robust errors; ^*^p < 0.10, ^**^p < 0.05, ^***^p < 0.01

Our results suggest that economic decision-makers are influenced by their emotions but are not slaves to them. Many individuals participate in MTurk first and foremost to earn money. Still, they are prone to emotional responses that depend on the framing of their choice environment. But it appears that they are able to exert some control over the transmission of emotions onto choice. We presume that the strength of such attenuation is moderated by financial need and largely absent in our field experiment.

## Conclusions

In this paper, we presented results from a field experiment on crowdfunding. We varied the message within the crowdfunding campaign in order to explore the role of donation and contribution frames. We found that a donation frame attracted more and higher gifts than a contribution frame. We furthermore documented that the word ‘donation’ is connotated with voluntary action and charity and hence might be more effective in generating warm glow for a donor and stimulating a positive self-image. In contrast, a contribution appears to be perceived more as an obligation or duty. We found support for this interpretation in an additional experiment run on MTurk: the word ‘contribution’ generated relatively more negative emotions than the word ‘donation’ did.

We also documented some interaction patterns between the framing and other features of the crowdfunding campaign, notably the strong attraction of giving thresholds that are associated with rewards. As such, our paper adds to the nascent literature on crowdfunding by pointing to some relevant trade-offs. Suggestions and thresholds can exert a strong pull in a contribution frame, turning reward structures into a powerful instrument. On the other hand, a donation frame triggers less negative emotional valence and inspires more basic generosity. In practice, these forces will have to be carefully weighed against each other. Crowdfunding campaigns should be designed from a holistic perspective, and the optimal design may vary between different types of goods. In light of our two experiments, we posit that the benefits of emotional manipulation will be less pronounced for projects that relate to economic necessities than for those that relate to luxury goods or charitable projects. At the same time, projects for economic necessities may benefit more from attractive reward structures coupled with a contribution frame that maximizes the pull of reward thresholds.

From a policy perspective, our results echo Enachescu et al.‘s (2019) call to consider emotional responses in institutional design, with tax collection as their leading example. Our results confirm their insight that positive emotions can increase generosity, but subtle differences emerge. After all, we observed stronger effects when the good to be financed was perceived already as a common enterprise (the institute’s summer party) and not just a work environment (MTurk). This points to important interaction effects and raises the question as to whether a state household could be framed as positively and participatorily as the party in our study. Probably not—but fungibility aside, states do finance some goods that may be more immediately perceivable as participatory. Given the current Covid-19 pandemic, health care easily comes to mind. In order to sharpen this point, let us make a prediction for different approaches on financing health care during the pandemic. Let’s imagine that we want to finance, say, a new wastewater project and a new hospital (or, perhaps, upgrades like new pipes for the former and more nurses for the latter). We simply implement the central variation of the present study, that is, we ask either for donations or contributions. The conjecture emerging from putting our findings into context would be that the donation frame would perform better than the contribution frame, particularly so for the hospital project.

While our field experiment explores a crowdfunding setting, the fundamental explanation for our treatment effects—that different frames trigger different emotions—should apply also to other settings in which acts may be framed as either donations or contributions. Given the surprisingly large effect of our small variation and its sensitivity to the precise choice environment (with substantial attenuation in the semi-professional world of MTurkers), we imagine that there is still a wide range of opportunities to pursue in this area of research.

## Electronic supplementary material

Below is the link to the electronic supplementary material.

Supplementary Material 1

## Footnotes

1 These differences are, of course, very much stylized. In reality, there are many hybrid forms to be found. For example, charitable giving that benefits others may benefit everyone if everyone cares about the benefit being generated for others. Also, sometimes charities do offer (small) rewards for donations (see, for example, Falk 2007) or some form of social recognition as a reward (see, for example, Glazer and Konrad, 1996).

2 Crowdfunding has become a popular tool to raise money for projects, attracting investments of US$25 billion in 2015 alone (Massolution, 2015). Successfully funded projects include movies, video games, software, and appliances but also charitable projects, scientific research for rare genetic diseases, and museum projects. One of the most successful projects so far has been the video game Star Citizen, which surpassed US$288 million in contributions in 2019 (https://www.forbes.com/sites/mattperez/2019/05/01/exclusive-the-saga-of-star-citizen-a-video-game-that-raised-300-millionbut-may-never-be-ready-to-play/#5819cd155ac9, retrieved on 9 April 2020). But there are also many campaigns for small projects, notably for the arts and for local purposes. In Europe, the volume of donation-based crowdfunding grew from €22 to €53 million between 2015 and 2017 (Ziegler et al., 2019, p.33).

3 Smaller communities of this type are expected to have higher giving frequencies but remain understudied (Andreoni, 1988; Kessler and Milkman, 2018).

4 Note that we used the term ‘bonus’ instead of ‘match’ in the announcement e-mails.

5 Google Trends: worldwide searches for 2004–2017. The difference is 65% in the first case and only 36% in the second case when looking at the United States only.

6 Chlaß, Gangadharan, and Jones (2021) studied two frames in which they described the amount withheld by an intermediary in a charitable giving process either as corrupt behaviour (cheating) or as the intermediary charging a fee to cover administrative costs. They found no difference in the response of donors to those different frames.

7 An early experiment that manipulated emotions through video clips can be found in Kirchsteiger et al. (2006), who studied gift exchange. The same technique was employed in Ifcher and Zarghamee (2011), who show that mood affects time preferences. Capra (2004) instead used a memory elicitation task to manipulate mood in order to study the role of mood in dictator, ultimatum, and trust games.

8 The question arises as to whether such positive effects of positive emotions are in some contradiction to Antinyan and Asatryan’s (2020) finding that nudges emphasizing deterrence are more effective than nudges appealing to morale. In light of our paper, morale might be a two-edged sword emotionally, with contributions appealing to the dark side of musts and donations to the more positive identity aspect of morale.

9 The department of economics accounted for less than 10% of the staff in the experiment.

10 A buffet pledge meant that the individual committed to bringing food (usually cake or salad) to the party.

11 Only the first e-mail included a translation into the local language.

12 Some of the characteristics were corrected by hand, for example, to exclude a person who oversaw messages in a particular list without being a member of that group.

13 Note that, given a large number of characteristics and a limited sample size, our approach does not ensure that individuals within a block are equal according to all characteristics, but it does ensure that they only differ on a few dimensions. The order was chosen by giving priority to the characteristics deemed more important to achieve balance on those dimensions. Some categories have no overlap such that their order does not matter; for example, the data management unit and the group of doctoral students are entirely distinct.

14 The extent to which we increased power by blocking was difficult to determine ex ante. It depends on how well the giving behaviour is explained by the individual characteristics on which we blocked.

15 Of course, some indirect spillovers could have been at play in the form of social influence (if one person were to have announced to colleagues that they had given a particular amount, the colleagues might have followed suit). Although we cannot completely exclude this, we can show that there was no clustering over time by the group to which individuals belonged (see Fig. 3 in the Appendix).

16 Gifts from people involved in the design of the experiment were excluded from the analysis.

17 Due to a lack of time data for non-donors, we cannot control for the amount of money donated thus far relative to the threshold in these regressions, as is often done to study the completion effect. In regressions on the intensive margin, we find no completion effect. The timing of the gifts is driven rather by the first e-mail and the reminders.

18 The direct effects of suggestions are summarized in Appendix B. We find evidence in favour of higher non-binding suggestions similar to those observed in Adena, Huck, and Rasul (2014). A higher suggestion of €20, relative to the suggestion of €10, changed the distribution of gifts (generating more €10 gifts and fewer €5 gifts, and changing both the median and the mode) and increased the overall return, although not significantly. These results differ from experiments on gift grids in Reiley and Samek (2018) and Adena and Huck (2020), who found detrimental effects of higher grids. A potential explanation for these differences may be that suggestions are softer than grids and that higher gifts also go hand-in-hand with greater rewards in a typical crowdfunding campaign. Fig. 6 in the Appendix shows the exact distribution relative to the suggested amounts.

19 We do not count one gift in the donation frame, since although the person asked on the last match day for money transfer details, the transfer itself occurred only later.

20 We selected subjects located in the United States for participation.

21 Version 3.0, http://www.affective-sciences.org/en/gew/, retrieved on 16 February 2020 (for details, see Scherer, 2005; Sacharin, Schlegel, and Scherer, 2012; Scherer et al., 2013).
