# Supplementary material for: Voluntary ‘donations’ versus reward-oriented ‘contributions’: two experiments on framing in funding mechanisms
Source: Exp Econ. 2022 Jul 25;25(5):1399–417. doi: 10.1007/s10683-022-09759-6 (PMC9309996; doi:10.1007/s10683-022-09759-6)
Supplement: Supplementary file 1 — Supplementary Material 1 [file 10683_2022_9759_MOESM1_ESM.pdf]

Online Appendix for

Voluntary 'donations' versus reward-oriented 'contributions:' Two experiments on framing in funding mechanisms

Appendix A Additional tables and figures 2

Appendix B Suggestions of €10 and €20 10

Appendix C Individual characteristics and heterogenous treatment effects 12

Appendix D E-mail content in the field experiment 17

Appendix E Instructions in the MTurk experiment 22

Appendix A Additional tables and figures

Figure 2 Number of contributors by day

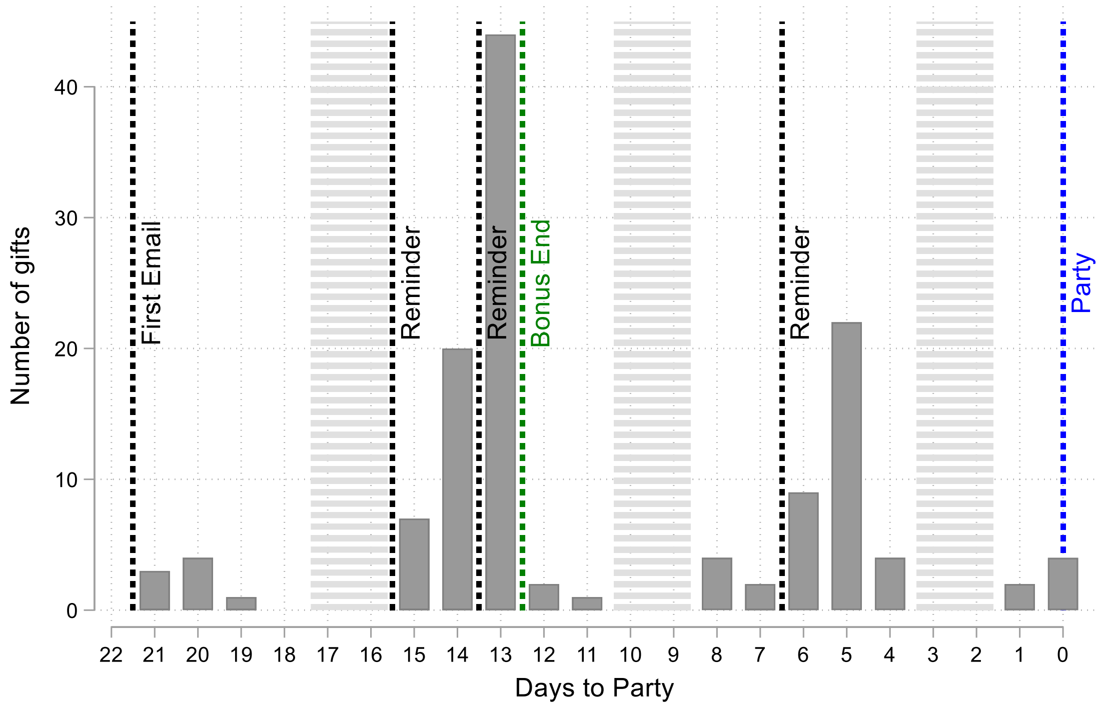

Figure 3 Number of gifts by group by day to party

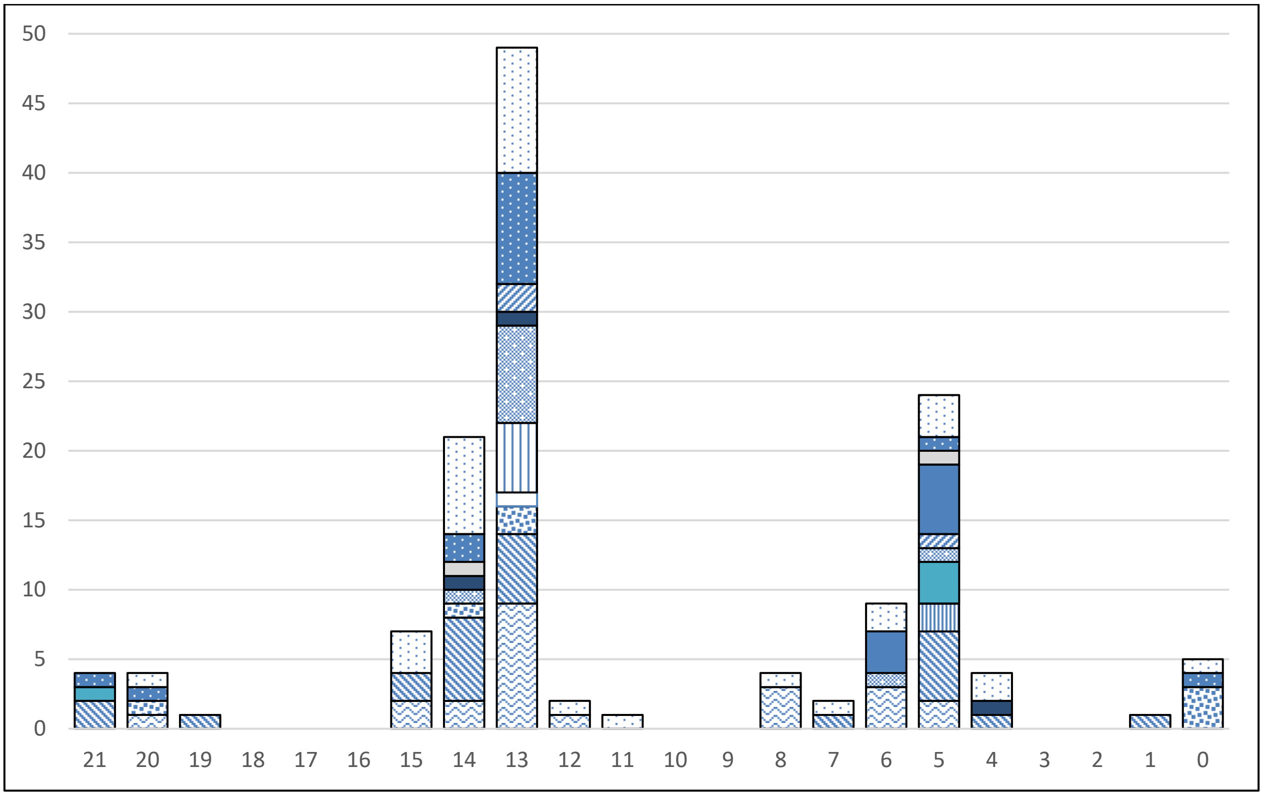

The figure above shows the numbers of gifts according to the institutional groups to which the contributors belonged (in 10 cases there is doubling as, for example, secretaries belong both to the administration and to their respective departments). For data protection reasons, we do not label the groups. We also do not correct for the size of the groups. While some groups cluster more around certain days, this does not appear to be a general pattern and may have occurred at random.

Figure 4 Associations with the words ‘contribution’ and ‘donation’ (source: http://www.snappywords.com/)

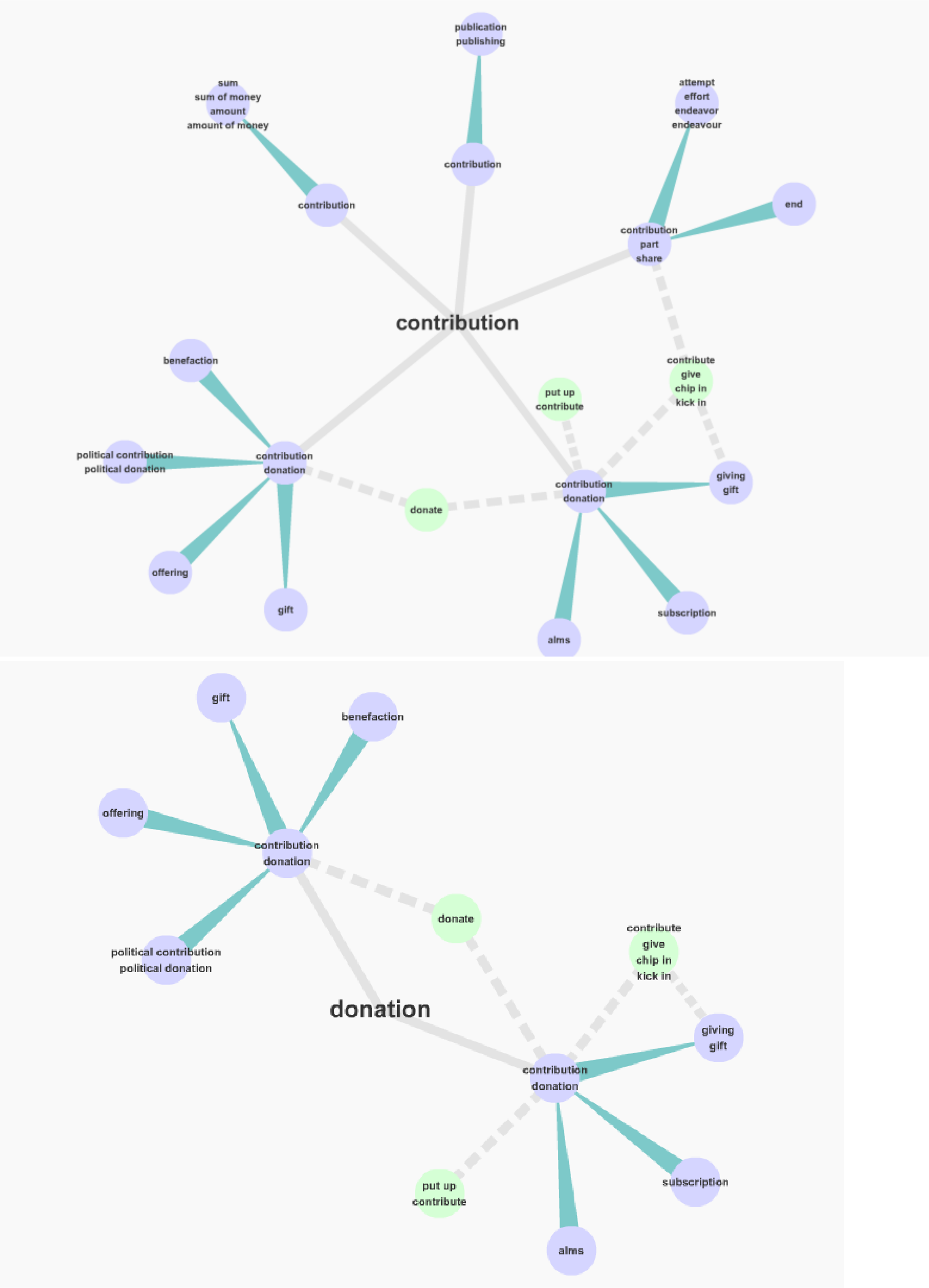

Figure 5 Fisherian randomization inference test for the treatment effect estimated in Table 2, Column I

Panel A: only monetary gifts

Panel B: including buffet pledges monetized at €10

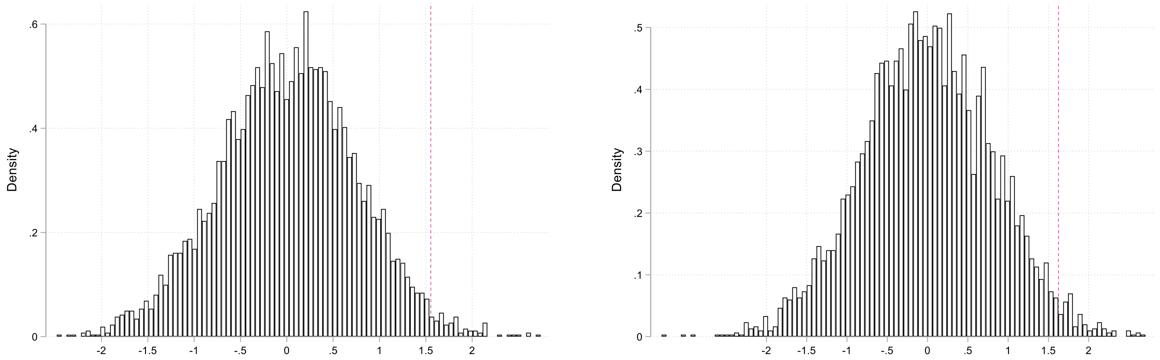

Note: Based on 5,000 permutations.

Table 7 Individual characteristics in each of the 2x2 randomization cells and t-test p-values

Treatment:	Donation				Contribution									
Treatment:	10€		20€		10€		20€							
	1		2		3		4		1=2	1=3	2=3	4=2	4=3	1=4
	N=135		N=137		N=137		N=136							
Share of participants pertaining to the group of:	Mean	Std. err.	Mean	Std. err.	Mean	Std. err.	Mean	Std. err.	Two-sided t-test p-values					
Females	0.504	0.043	0.511	0.043	0.504	0.043	0.511	0.043	0.905	0.999	0.904	0.953	0.951	0.952
Professors	0.044	0.018	0.051	0.019	0.051	0.019	0.051	0.019	0.798	0.798	1.000	0.798	0.798	1.000
Postdocs	0.141	0.030	0.139	0.030	0.146	0.030	0.139	0.030	0.961	0.902	0.863	0.898	0.764	0.860
PhD students	0.207	0.035	0.182	0.033	0.190	0.034	0.182	0.033	0.606	0.717	0.877	0.832	0.953	0.762
Student research assistants	0.141	0.030	0.153	0.031	0.161	0.031	0.153	0.031	0.771	0.649	0.869	0.906	0.778	0.863
Faculty I	0.185	0.034	0.204	0.035	0.212	0.035	0.204	0.035	0.691	0.585	0.882	0.951	0.931	0.647
Faculty II	0.081	0.024	0.102	0.026	0.095	0.025	0.102	0.026	0.556	0.698	0.840	0.710	0.865	0.828
Faculty III	0.126	0.029	0.117	0.028	0.117	0.028	0.117	0.028	0.818	0.818	1.000	0.450	0.450	0.328
Faculty IV	0.074	0.023	0.044	0.018	0.051	0.019	0.044	0.018	0.291	0.436	0.777	0.979	0.798	0.304
Faculty V	0.096	0.025	0.124	0.028	0.124	0.028	0.124	0.028	0.466	0.466	1.000	0.964	0.964	0.440
Administration	0.081	0.024	0.080	0.023	0.058	0.020	0.080	0.023	0.971	0.458	0.477	0.971	0.458	1.000
IT	0.007	0.007	0.015	0.010	0.015	0.010	0.015	0.010	0.571	0.571	1.000	0.571	0.571	1.000
Library	0.030	0.015	0.022	0.013	0.044	0.018	0.022	0.013	0.689	0.536	0.311	0.689	0.536	1.000
Press unit	0.022	0.013	0.022	0.013	0.015	0.010	0.022	0.013	0.986	0.642	0.653	0.986	0.642	1.000
Secretaries	0.059	0.020	0.073	0.022	0.066	0.021	0.073	0.022	0.650	0.827	0.813	0.650	0.827	1.000

Note: All variables above are dummy variables; there are overlaps between the different categories. Std. err.: standard errors.

Table 8 Early and late gifts

	Early gifts with match offer of €5					Late gifts without match offer				
Treatment	Total number of gift givers	Buffet pledges	Number of monetary gifts	Average positive monetary gift	Average positive gift including buffet pledges monetized	Total number of gift givers	Buffet pledges	Number of monetary gifts	Average positive monetary gift	Average positive gift including buffet pledges monetized
	I	II	III	IV	V	VI	VII	VIII	IX	X
Contribution	36	12	33	8.33<br>(0.891)	11.67<br>(0.976)	25	4	22	9.44<br>(1.359)	11.04<br>(1.575)
Donation	43	13	40	12.44<br>(2.531)	15.47<br>(2.439)	25	5	22	14.4<br>(3.898)	16.4<br>(3.759)

Table 9 Google Trends searches worldwide (01.01.04–15.12.17)

**Donation**	**Relative frequency**	**Contribution**	**Relative frequency**
blood	100	ira	100
blood donation	95	ira contribution	100
organ donation	45	401k contribution	75
plasma donation	40	401k	75
plasma	35	what is contribution	55
donate	35	roth contribution	50
goodwill	25	ira contribution limits	50
donation center	25	roth ira	40
goodwill donation	25	roth ira contribution	40
egg donation	20	contribution margin	35
donation letter	20	sss	35
sperm donation	20	sss contribution	30
salvation army donation	20	hsa contribution	25
salvation army	20	hsa	25
donation request	20	401k limits	25
charity donation	20	401k contribution limits	25
red cross donation	20	roth contribution limits	25
donation pick up	20	cpf	25
red cross	15	cpf contribution	20
car donation	15	roth ira contribution limits	20
hair donation	15	defined contribution	20
clothing donation	15	maximum 401k contribution	20
furniture donation	15	lotto contribution	20
red cross blood donation	10	lotto world contribution	15
clothes donation	10	contribution definition	15

Table 10 Examples of the use of the word ‘contribution’ by charities and projects at a crowdfunding platform

Charity	Citation	Context	Source
Panel A: Examples from charities’ own websites			
American Red Cross	Charitable **Contributions**. Donations to the American Red Cross are tax deductible to the full extent of the law.	Tax treatment	https://www.redcross.org/donations/ways-to-donate/charitable-contributions.html
UNICEF	Sweden **contributes** US$2.7 million to UNICEF’s emergency response for children in Syria	Government donations	https://www.unicef.org/mena/press-releases/sweden-contributes-us27-million-unicefs-emergency-response-children-syria
United Way	When you give to United Way, your **contribution** helps foster both individual and collective success.	Individual donations	https://www.unitedway.org/get-involved/ways-to-give#
APOPO	APOPO Cambodia is deeply grateful for the support and generous **contributions** of its partners and donors.	Corporate donations	https://www.apopo.org/en/latest/2020/12/APOPO-and-CMAC-commit-to-another-year-of-partnership
DNDi	Listed below are supporters who have given a cumulative **contribution** of over USD or EUR 10,000 since 2003, as well as collaborative funding partners.	Government donations	https://dndi.org/about/public-donors/
Oxfam UK	In 2010/11, more than 40 institutional donors **contributed** an all-time high of £173.5 million to our projects worldwide.	Institutional donations	https://www.oxfam.org.uk/about-us/how-we-work/about-our-partners/
Tree of Hope	you can help by **contributing** to that campaign fund	Individual donations	https://www.treeofhope.org.uk/ways-to-donate/donate-to-a-campaign/
Unseen	Just set up a Just Giving page for your friends and family to pay in their **contributions** – or pay in lump sums easily yourself.	Individual donations	https://www.unseenuk.org/get-involved/helpline-hero/
Safe Line	Donate to us. … Your **contribution** can change lives for the better.	Individual donations	https://www.safeline.org.uk/support-us/why-support-us/
Panel B: Examples from project descriptions on an online crowdfunding platform			
GoFund Me	Ramadhan gives each and every one of us the opportunity to **contribute** to charity and be a part of uniting our Ummah.	Individual donations	https://www.gofundme.com/f/7rbym-gift-of-water
GoFund Me	please consider **contributing** to this sweet family	Individual donations	https://www.gofundme.com/f/sza4d-family-in-need-due-to-covid19
GoFund Me	**Contribute** to Lifesaving Medical Care in Lebanon	Individual donations	https://www.gofundme.com/f/contribute-to-lifesaving-medical-care
GoFund Me	If anyone would like to **contribute** to our funds please see link below.	Individual donations	https://www.gofundme.com/f/p2p29z-kindness-homeless-street-team-glasgow
GoFund Me	We would love for your support by making a **contribution** to the 2nd annual Staff Appreciation Fund.	Individual donations	https://www.gofundme.com/f/ghes-staff-appreciation-fund
GoFund Me	I would be very grateful if anyone is able to **contribute**.	Individual donations	https://www.gofundme.com/f/help-with-orthopedic-surgery-in-kenyan-hospital

Table 11 MTurk survey and emotion levels by frame

	Donation		Contribution		
	N= 474		N= 511		
	Mean	Std. err.	Mean	Std. err.	t-test p-value
Interest	63.015	1.274	67.456	1.186	0.011
Amusement	35.565	1.479	41.941	1.425	0.002
Pride	46.219	1.587	47.840	1.463	0.453
Joy	48.276	1.504	47.182	1.412	0.596
Pleasure	50.173	1.463	51.002	1.376	0.680
Contentment	53.479	1.504	55.996	1.352	0.214
Love	37.928	1.550	34.082	1.452	0.070
Admiration	33.850	1.490	34.070	1.394	0.914
Relief	28.992	1.372	33.098	1.323	0.031
Compassion	49.105	1.573	42.965	1.457	0.004
Sadness	7.274	0.704	9.634	0.764	0.023
Guilt	9.439	0.804	12.260	0.882	0.018
Regret	9.338	0.708	12.759	0.884	0.003
Shame	7.968	0.738	10.630	0.850	0.018
Disappointment	7.561	0.655	10.487	0.822	0.005
Fear	8.063	0.700	11.992	0.845	0.000
Disgust	5.589	0.555	8.667	0.773	0.001
Contempt	12.447	1.064	15.415	1.089	0.052
Hate	5.361	0.534	7.159	0.683	0.038
Anger	5.411	0.545	7.675	0.700	0.011

Note: Std. err. = standard errors.

Although we were unable to determine exact participation at the party, it seemed to be similar to previous years. Below, we present the numbers of people who donated, those who were eligible to take part in games, and those who actually took part in the games.

Table 12 Participation at the party

Donated €5 or more	Donated at least €10 or buffet	Donated at least €20 or buffet+€10				
Eligible for participation in games or more	Eligible for only 1 experiment	Eligible for 2 experiments	Maximum possible participation in experiments	Actual participation in experiment 1	Actual participation in experiment 2	sum
130*	57	34	125	49	28	77

Note: * The exact number of attendees is unknown, though we estimate it to be larger than 130. Some guests brought family members; some employees joined for a short time and went back to work; some came early and left early, while others came and left late. Given the many points of entry and exit and different timings, it was not possible to count the number of attendees. Games and experiment 1 were organized in the form of stations, while experiment 2 took place at one point in time. Not all eligible participants took part in the experiments for various reasons, for example, timing or preferring to chat with others.

Appendix B Suggestions of €10 and €20

Table 13 Results of suggestions

Treatment	€ 10		€ 20		T-test p-value	Test of proportions p-value
Panel A: only monetary gifts						
Number of subjects	272		273			
Number of monetary gifts	61		59			
Share of monetary gifts	0.224	(0.025)	0.216	(0.025)		0.8185
Monetary return per mail in €	2.5	(0.472)	2.788	(0.508)	0.679	
Average positive monetary gift in €	11.148	(1.699)	12.898	(1.833)	0.485	
Minimum in €	5		5			
Median in €	5		10			
Maximum in €	100		100			
Share of gifts €5–6 conditional on giving	0.508	(0.064)	0.322	(0.060)		0.0386
Share of gifts €10 conditional on giving	0.279	(0.057)	0.424	(0.064)		0.0958
Share of gifts €15 and more conditional on giving	0.213	(0.052)	0.254	(0.057)		0.5944
Panel B: including buffet pledges monetized at €10						
Number of buffet pledges	18		16			
Share of buffet pledges	0.066	(0.015)	0.059	(0.014)		0.7149
Total number of gift givers	66		64			
Overall response rate	0.243	(0.026)	0.234	(0.026)		0.8220
Return in € per mail including buffet pledges monetized at €10	3.162	(0 .515)	3.374	(0 .539)	0.776	
Average positive gift in € including buffet pledges monetized at 10€	13.030	(1.605)	14.391	(1.686)	0.560	

Note: standard error in parenthesis

Figure 6 Frequency of different gift values by donation/contribution frame and different suggestions.

Panel A: only monetary gifts
Panel B: including buffet pledges monetized at €10

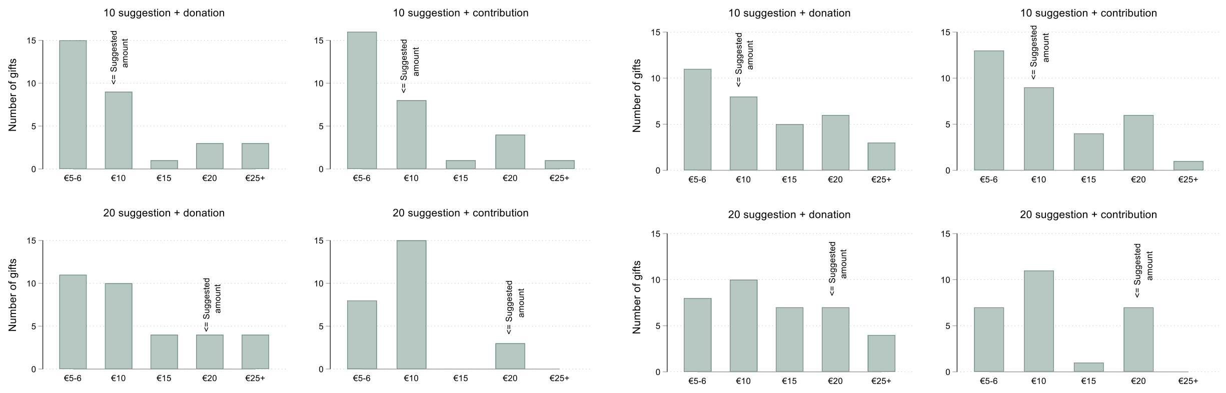

Table 13 presents the results by different suggestion levels. While the response rate was almost identical in both treatments, the average positive monetary gift increased by €1.75 or 16% when the higher amount was suggested (not significant). The median increased from €5 in the €10 suggestion treatment to €10 in the €20 suggestion treatment. Since the shares of individuals that contributed to the buffet were similar between the two treatments, we do not see any substitution between monetary and non-monetary donations. Figure 6 presents the distribution of different gift categories by the suggested level (€10 and €20) and frame. There is a visible shift in the distribution towards larger amounts with higher suggestions. Moreover, the mode increases from €5 with lower suggestions to €10 with higher suggestions. Table 13 confirms the impression from Figure 6. The giving frequency of €5 is higher with lower suggestions, and this difference is statistically significant. The giving frequency of €10 as well as that of €15 and up are higher with higher suggestions, though only the first difference is statistically significant. While the overall monetary return is higher with higher suggestions, it is so only by 12%, and this difference is not statistically significant.

Appendix C Individual characteristics and heterogenous treatment effects

In this section, we explore the available information on the personal characteristics of the participants in our field experiment. However, one must be cautious with the interpretation, since these characteristics are likely related to the actual attendees of the summer party and this, in turn, with participation in the crowdfunding campaign.

In Table 14, we present the results from simple regressions including individual characteristic dummies.[1] Column I shows the monetary return per e-mail by presenting the results from an OLS regression with monetary gifts (including zeros) as the dependent variable. Column II shows the effect of individual characteristics on positive gifts only (OLS regression). Column III analyses the response rate by presenting the marginal effects from a Probit regression. When looking at the dummies professor, postdoc, PhD student, student research assistants, and administrative staff, note that the reference group is the remainder including current guests, alumni, and affiliated researchers not on the institute's payroll. First, we see that the response rate of postdocs, PhD students, and administrative staff is significantly higher. In terms of positive gifts, those given by professors clearly stand out (an increase by €30). The positive gifts by student research assistants are significantly lower (by almost €6). The combined result—the return—is significantly lower for student research assistants.

Next, we present separate and more detailed comparisons between the group of academics and the administrative staff, subgroups of the academics only, and between male and female e-mail recipients that confirm the above results. We also tested for heterogeneous treatment effects and found that females responded more often when the donation framing was used and that the administrative staff members were less responsive to higher suggestions.[2]

[1] We did not control for block fixed effects here because this would only be feasible in Column I.

[2] We chose gender and administrative status for the heterogeneity analysis since this divides the sample into relatively large groups. Gender differences in positive versus negative frames in public good games have been studied by Fujimoto and Park (2010), who found that gift levels are similar for both genders in the positive frame, while male subjects give significantly lower amounts in the negative frame. With our interpretation of the donation frame being more positive, our results differ from Fujimoto and Park (2010). The results in Table 17 suggest that female participants gave significantly more often than males in the donation frame, but this might have been driven by more females working in administration and thus having lower incomes as well as by higher participation from the administrative staff, which seems in line with the results in Table 18.

Figure 7 shows the average returns in both frames by academic status. This status also corresponds to large income (also age) differences. While in the contribution frame, the gifts seem not to be strongly related to status/income, they are in the donation frame.[3]

Table 14 Individual characteristics

	Monetary return	Average positive gift	Overall response rate
	OLS	OLS	Probit marginal effects
'Donation'	1.402**	4.265*	0.030
	(0.676)	(2.311)	(0.036)
€20 suggestion	0.189	1.604	-0.013
	(0.692)	(1.867)	(0.036)
Female	0.229	-2.576	0.039
	(0.880)	(2.848)	(0.037)
Professor	6.394	30.731**	0.023
	(4.252)	(13.823)	(0.090)
Postdoc	1.327	-2.405	0.148***
	(0.837)	(2.290)	(0.055)
PhD student	0.528	-3.239*	0.114**
	(0.644)	(1.731)	(0.051)
Student research assistant	-1.424***	-5.887***	-0.092
	(0.465)	(1.598)	(0.064)
Administrative staff	1.815	1.111	0.154***
	(1.178)	(2.800)	(0.048)
Constant	0.929	10.293***	
	(0.830)	(2.762)	
Observations	544	119	544
R^2/ Pseudo R^2	0.050	0.280	0.044

Note: Robust standard errors in parentheses; not accounting for buffet contributions; * $p < 0.10$, ** $p < 0.05$, *** $p < 0.01$.

[3] Note that there might not be that large of a difference in income between postdocs and doctoral students. These individuals are usually remunerated according to the same pay scale, but doctoral students often hold less than full-time (typically 66–75%) contracts.

Figure 7 Average unconditional gifts by status

Only monetary gifts Including buffet pledges monetised at €10

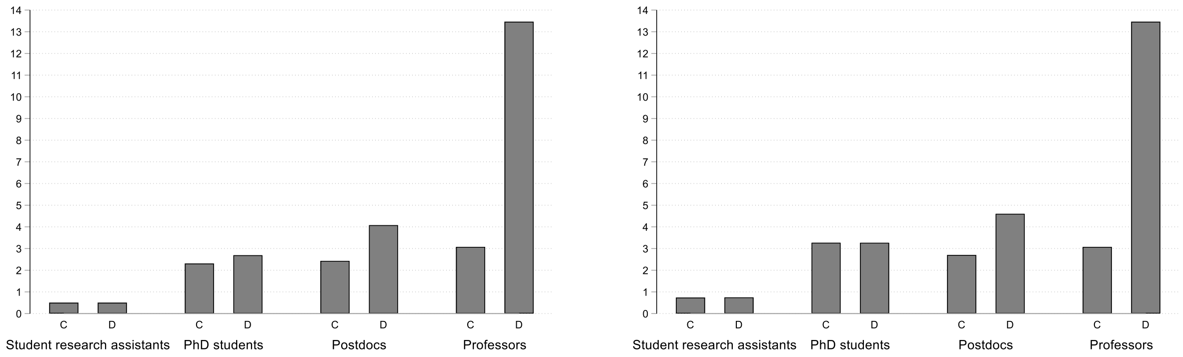

Note: C – treatment ‘contribution’, D – treatment ‘donation’

Table 15 Academics versus administration

Group	Number of subjects	Number of monetary gifts	Overall return per e-mail	Average positive gift	Minimum Median Maximum	Share monetary gift	Share buffet	Overall response rate
Academics	325	64	2.354<br>(0.429)	11.953<br>(1.731)	5<br>10<br>100	0.200<br>(0.022)	0.046<br>(0.012)	0.203<br>(0.022)
Administration	118	36	3.686<br>(.958)	12.083<br>(2.675)	5<br>10<br>100	0.331<br>(0.033)	0.085<br>(0.026)	0.339<br>(0.044)
T-test p-value			0.147	0.966				
Test of proportions						0.004	0.120	0.003

Note: Standard errors in parentheses.

Table 16 Gender

Group	Number of subjects	Number of monetary gifts	Overall return per mail	Average positive gift	Minimum Median Maximum	Share monetary gift	Share buffet	Overall response rate
Male	269	54	2.494	12.426	5	0.204	0.048	0.212
			(0.485)	(1.899)	10	(0.025)	(0.013)	(0.025)
					100			
Female	276	66	2.790	11.667	5	0.261	0.076	0.264
			(0.496)	(1.660)	10	(0.026)	(0.016)	(0.027)
					100			
T-test p-value			0.670	0.763				0.150
Test of proportions						0.119	0.180	0.150

Note: Standard errors in parentheses.

Table 17 Interaction with gender

	Monetary return	Average positive gift	Overall response rate
	OLS	OLS	Probit marginal effects
‘Donation’	0.683	6.283	-0.053
	(0.976)	(4.324)	(0.053)
€20 suggestion	-0.720	-2.252	-0.037
	(0.972)	(3.753)	(0.053)
Female	-1.362	-3.586	-0.053
	(0.912)	(2.861)	(0.063)
Female x ‘donation’	1.337	-2.698	0.155^{**}
	(1.384)	(5.152)	(0.072)
Female x €20 suggestion	1.969	6.918	0.053
	(1.377)	(4.917)	(0.073)
Constant	2.513^{***}	10.676^{***}	
	(0.687)	(2.102)	
Observations	545	120	545
R^2 /Pseudo R^2	0.013	0.052	0.013

Note: Robust standard errors in parentheses; * $p < 0.10$, ** $p < 0.05$, *** $p < 0.01$.

Table 18 Interaction with administrative staff

	Monetary return	Average positive gift	Overall response rate
	OLS	OLS	Probit marginal effects
‘Donation’	1.256*	4.830*	0.017
	(0.704)	(2.562)	(0.042)
€20 suggestion	1.074	3.630	0.011
	(0.707)	(2.586)	(0.042)
Administrative staff	3.086**	3.576	0.137*
	(1.364)	(2.806)	(0.073)
Administrative staff x ’donation’	0.787	0.047	0.062
	(2.015)	(6.048)	(0.084)
Administrative staff x €20 suggestion	-4.116*	-7.735	-0.099
	(2.110)	(5.825)	(0.083)
Constant	1.160**	7.395***	
	(0.471)	(1.766)	
Observations	544	119	544
R^2	0.024	0.049	
Pseudo R^2			0.018

Note: Robust standard errors in parentheses; * $p < 0.10$, ** $p < 0.05$, *** $p < 0.01$.

Appendix D E-mail content in the field experiment

First email (Different versions are marked with curly and angle brackets)

Dear (name of the institute)-ers and friends,

This year our (name of the institute) summer party follows the motto

There is such a thing like a free lunch.

The party will take place on Tuesday, the 5th of July, beginning at 4pm.
And so this time we do not want to install a cash box on the day, **however we do need your contributions {donations} to a crowdfunding campaign now**. Below you will find more information.

The (department name) is planning a party with:

[Food & Drinks]: We are planning a BBQ with organic sausages that come from appropriately treated animals as well as the usual assortment of alcoholic and non-alcoholic beverages. In addition, there will be the well renowned (name of the institute) potluck buffet of salads and cakes.

[Special Entertainment]: We are planning several (team) games and hands-on experiments, music, as well as a small campfire. Childcare and fun activities for children will be organized as usual by the Family Service.
As usual, please send the information regarding the number of children for whom you need child care, and their respective ages to: (e-mail address) by June 24, 2016.

In order to ensure that it will be a wonderful party, we are now starting a

>>>>>>>>>> **Crowdfunding Campaign** <<<<<<<<<<<

Contribute {Donate} to our summer party, please!

For our summer party, we need your support with the food and drinks. You can do this through in-kind or money contributions, or preferably both!
So, please, prepare salads and bake cakes for the 5th of July, and please also open your wallet (now)!

For each contribution {donation} there is a **Thank You**, staggered as follows:

[from € 5]:
- 1 pass for all games and competitions (for example, Kicker, Kubb, Ping Pong)

[from € 10 or 1 buffet contribution {donation}]:
- 1 pass for all games and competitions (for example, Kicker, Kubb, Ping Pong)

- Participation in a decision experiment with the possibility of winning 50 Euros or Participation at a ‘tasting station’ with the possibility of winning 50 Euros

[from € 20 or € 10 +1 buffet contribution {donation}]:
- 1 pass for all games and game competitions (for example, Kicker, Kubb, Ping Pong)
- Participation in a decision experiment with the possibility of winning 50 Euros
- Participation at a ‘tasting station’ with the possibility of winning another 50 Euros

[from € 30 or € 20 +1 buffet contribution {donation}]:
- 1 pass for all games and game competitions (for example, Kicker, Kubb, Ping Pong)
- Participation in a decision experiment with the possibility of winning 50 Euros
- Participation at a ‘tasting station’ with the possibility of winning another 50 Euros
- We will play 5 songs of your choice

[over 100 € or 90 € + 1 buffet contribution {donation}]:
- 1 pass for all games and game competitions (for example, Kicker, Kubb, Ping Pong)
- Participation in a decision experiment with the possibility of winning 50 Euros
- Participation at a ‘tasting station’ with the possibility of winning another 50 Euros
- We will play 5 songs of your choice
- A copy of the book ‘Fleisch und Farbe’ (unique limited edition book, comprising only 100 individually numbered prints).

For every contribution {donation} made before 22.06.2016, an anonymous sponsor will make a **bonus contribution {donation} of € 5** on your behalf. (However, these 5 euros are not included in the calculation of your ‘Thank You’ Coupon.)

If the average monetary contribution {donation} is **20 € <10€>**,
we need **100<200>** participants in the campaign
to cover the expected costs.*

The current status of contributions {donation} will be documented daily on the Intranet at (web address) (right column, updated each afternoon at 5 o'clock, Friday at 3).

Your generous monetary contributions {donation} (or willingness to contribute {donation} to the buffet) can be confidentially made to (name) (room (number), between 9am-12 and 1pm - 5pm). (*Those who cannot make the contribution {donation} in person may contact (name) [at: (e-mail address)] for the account details in order to do an online bank transfer*) **

[Your contribution {donation} does even more!]: Your contribution {donation} doesn’t only support the summer party as a public good. If we receive more contributions {donation} than required for financing the party, then the surplus will be used for an additional worthy project, e.g. to support the Women’s Bike Project, facilitated by the AG Refugees.

We look forward to your active participation in the crowdfunding campaign and, also, to a great party,

The (department name)

* The revenues will also be used to cover various minor costs, such as the purchase of bread, rolls, paper plates and cutlery as well as the music organization.

** We will not announce any individual contribution {donation} information and guarantee confidentiality.

****

First reminder

Dear (name of the institute)-ers and friends,

Maybe you have overlooked our email last week starting a **crowdfunding campaign** for this year's **summer party** (see below). We really believe that a party is much nicer without cash boxes so we hope you will join the crowd and help fund the party.

Remember that if you contribute {donate} this week **until Wednesday** it will generate a **match** from an anonymous benefactor of **five additional euros**.

All best

The (name of the institute) Party Team

P.S. Crowdfunding barometer can be seen at (web address)! Take a look!

****

Second reminder

Re: Last match day ((name of the institute)summer party 2016)

Dear (name of the institute)-ers and friends,

while our crowdfunding campaign for the summer party will continue until **end of June**, **TODAY** is the last day where every contribution {donation} that we get will be matched by an additional **5 €** from an anonymous benefactor.

Until yesterday we collected inspiring **495€ (+185€ Boni) + 16 buffet pledges.**
Many thanks to all contributors {donors} so far!
However, we are far away from the threshold we aim at
(Needless to say, it won’t even cover the drinks).
Therefore, we need you to

join the crowd now!

To clarify all open questions, let us explain the purpose and working of this campaign once more:
Everything what was traditionally organized and more: food (including vegetarian burgers and organic sausages), drinks (alcoholic and non-alcoholic), as well as music **WILL BE FREE** on the day. In addition, there will be the well renowned WZB potluck buffet of salads and cakes (also FREE).

The rewards offered within the crowdfunding campaign are made only possible by the additional efforts of our department, are by no means standard, and should serve as additional motivation for the participation in the crowdfunding campaign.

Follow the progress of the campaign at (web address)

All best
The (name of the institute) Party Team
****

Third reminder

Last call: summer party crowdfunding and program

Dear (name of the institute)-ers and friends,

Less than a week is left till our amazing (name of the institute) summer party 2016 which takes place on Tuesday, **5th of July, starting at 4 p.m**. Since we don't have a huge external sponsor this year, we need to rely on your participation in the **crowdfunding campaign** to finance the party!

Until yesterday we collected inspiring 980€ (+395€ Boni) + 25 buffet pledges.
Many thanks to all contributors {donors} so far!
However, **we are still missing the threshold** we aim at.
Two days left for contributions {donations}!
Therefore, we need you to
join the crowd now!
(contributions {donations} are collected till the end of June by (name),
Room (number), 9-12 a.m. and 1-5 p.m.)

Remember: If the average monetary contribution {donation} is **20 €<10€>**,
we **need 100<200> participants** in the campaign
to cover the expected costs.

Last call: please send the information regarding the number of children you would like to sign in for the **(name of the institute) Kinderfest** (organized by Familienservice child care animators),

and their respective ages **TODAY** to: (e-mail address).

Preliminary program:

From 4:00 p.m.	**Barbeque** (including veggie and vegan options), **drinks**, and **potluck buffet**
From 4:00 p.m.	**(name of the institute) Kinderfest** fun activities for children.
4:00-5:30 p.m.	**Tasting experiment** (Provided you are eligible, you may participate at any time while open. It won't take long, and you have the chance of winning 50 Euros.)
From 4:00 p.m.	**Tournaments** (in order to take part in Kicker (Foosball) or Table Tennis (Ping Pong) tournament you must sign up (alone or in pairs) till Friday 2 July with (e-mail address). You will be assigned the staring time. Kubb will be open for spontaneous teams.)
5:00 p.m.	Experiment 2 (Those who are eligible will get a separate Email with instructions. It is necessary to be on time since the experiment takes place simultaneously for all participants. You must also bring either your smart phone, tablet or laptop with an internet connection with you. There is a chance to win 40 or 10 Euros.)
5:30 p.m.	We play your songs
6:00 p.m.	The results and winners of the experiments will be announced
6:30-8:00 p.m.	We are pleased to announce that 8name) and his band **(name)** (web address) will play at our party
6:30 p.m.	**Long drinks** stand will be opened

Follow the progress of the crowdfunding campaign at (web address)

All best

The (name of the institute) Party Team

Appendix E Instructions in the MTurk experiment

Start page:

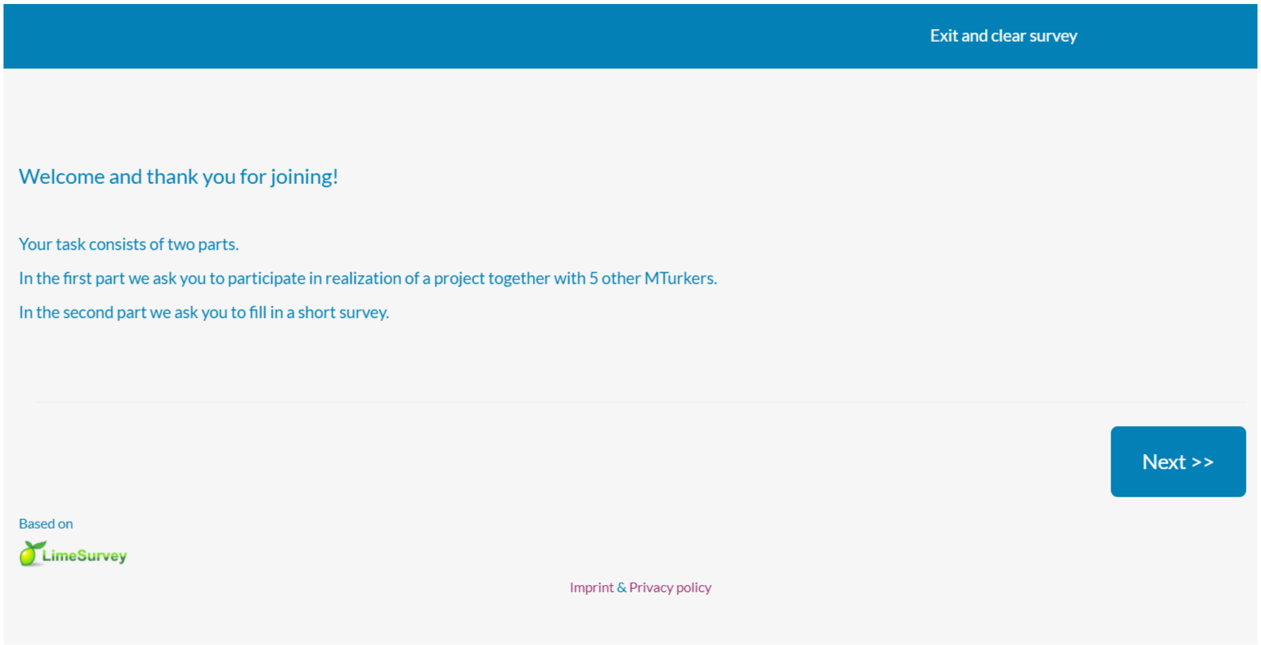

On the page below, participants were shown instructions and decided on the amount of their gift to the joint account. The screenshot shows the contribution frame. In the donation frame, the term 'contribution' was exchanged for the word 'donation'. There was no prespecified (default) position of the slider: the blue indicator and explanation below only appeared once the individual had clicked. Participants could adjust the slider until choosing their preferred position.

Exit and clear survey

0%

Part 1 - Project

Task

In this part you and 5 other anonymous MTurkers are matched together and receive exactly the same instructions. Each of you receives a budget of $2. We ask you to contribute to a joint project in order to make it successful.

Contributions towards a group account

You can contribute any amount between 0 and 2$.

Any amount you keep will be directly added to your final payment, in addition to the base payment of 50c, and any bonus that may result from the project.

The amount you contribute will help to make the project successful.

Project success

Your contribution and the contributions of the other 5 participants will be payed towards a group account. The project will be successfully realized if the total group contributions are at least $5. If the project is realized, the money in the group account will be **doubled**.

If the total group contributions fall short of $5, the project will not be realized, and the money in the group account will not be doubled.

In both cases the money will be equally distributed among all group participants regardless of their own contributions.

Rewards

In order to thank you for your participation in the project we have designed additional rewards depending on the level of your contribution:

If you contribute at least 20c :

- a bronze contributor recognition award.

If you contribute at least 40c :

- a silver contributor recognition award
- reward of 5c added directly to the final payment.

If you contribute at least 80c :

- a golden contributor recognition award
- reward of 10c added directly to the final payment.

If you contribute at least 1.6$:

- a diamond contributor recognition award
- reward of 20c added directly to the final payment.

Please choose your contribution (centwise):

Your contribution

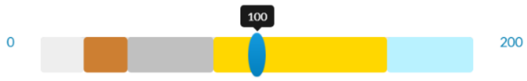

For your contribution of 100 cents,
you will get the golden award
and an additional reward of 10 cents.

Next >>

Next, participants received a symbolic award provided that they met one of the thresholds. The next screenshot shows an example of the silver donor award in the donation treatment.

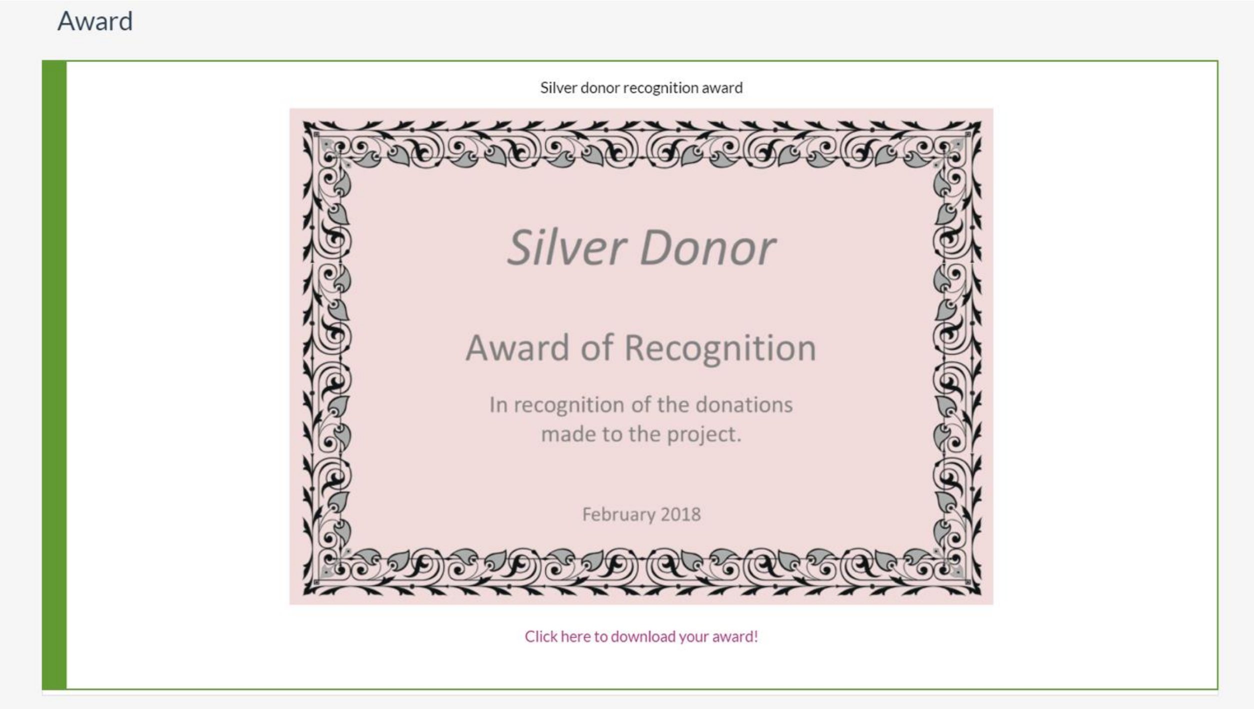

Next, participants were asked how they felt when making their donation/contribution. They were presented with all 20 emotions from the GEW in a random order and marked their responses by moving the slider (again, there was no prespecified position). The screenshot below shows an example with two emotions in the donation frame (the emotion questions were shown in random order).

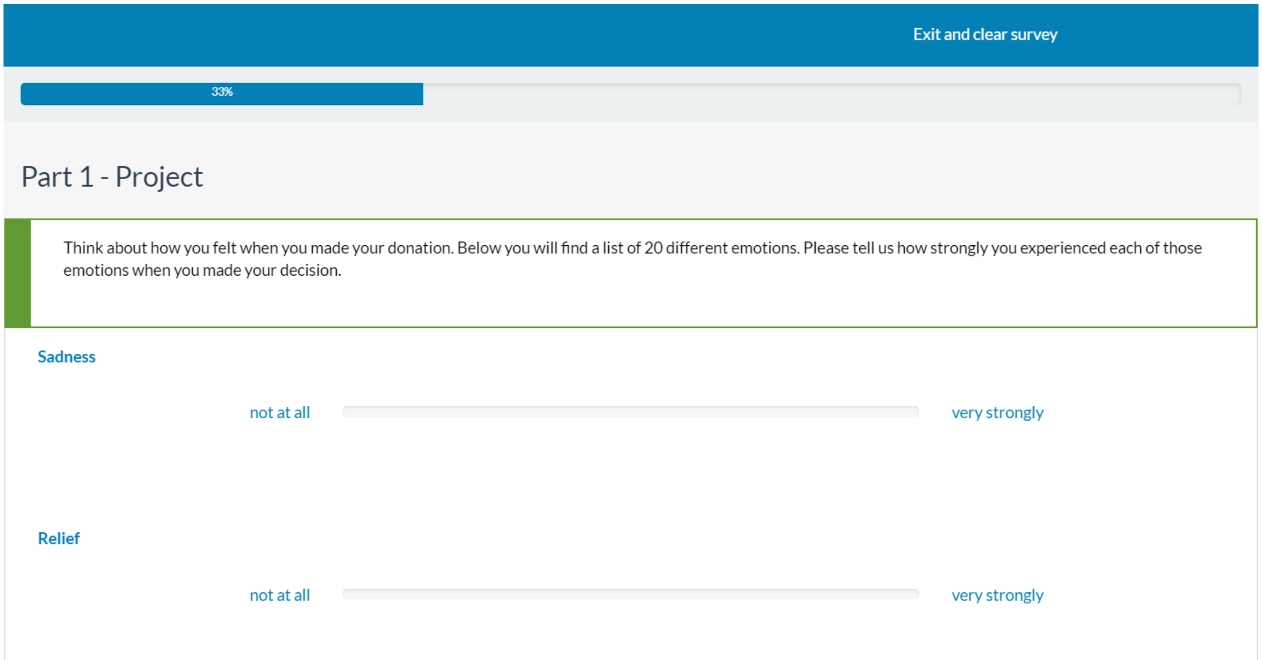

Next, participants were asked what they thought regarding the level of donations/contributions by other participants in their group, on average.

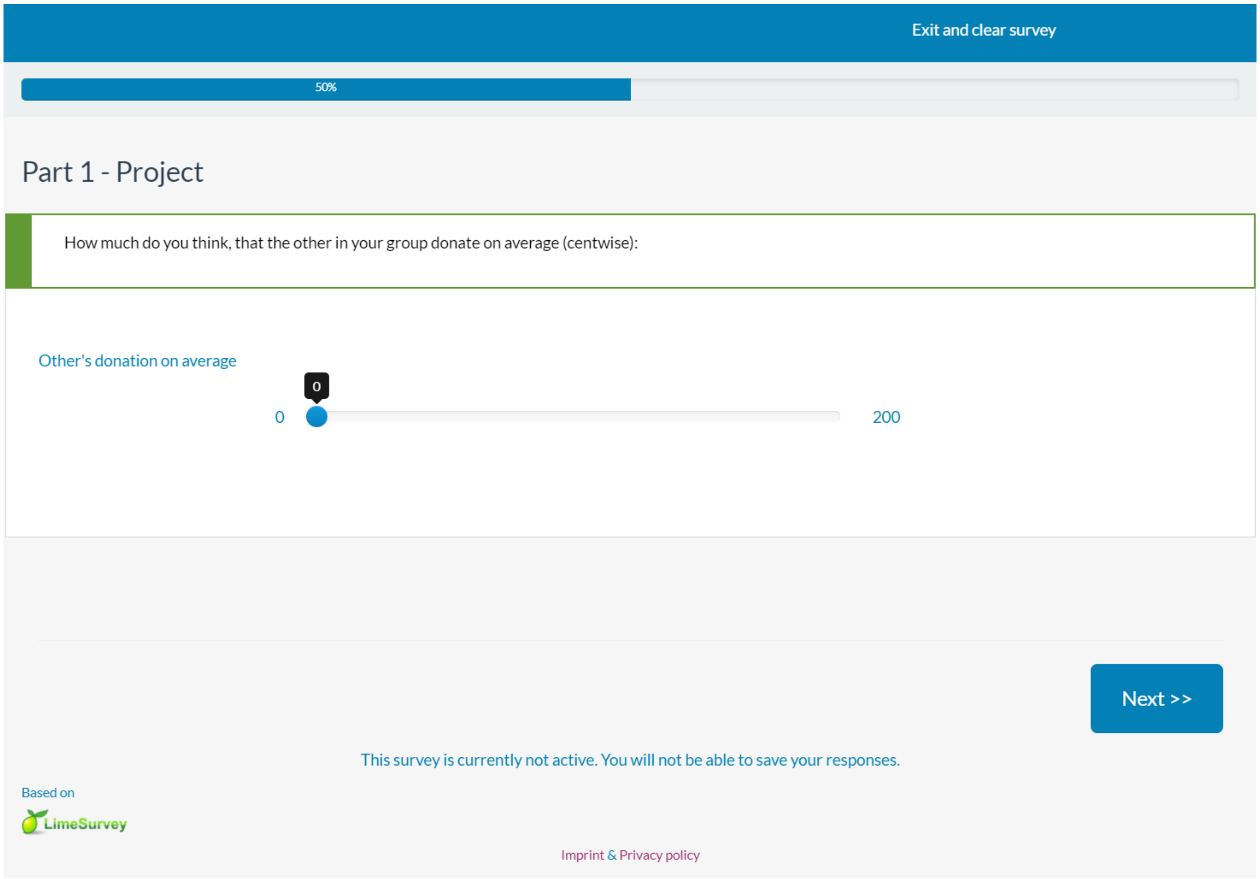

Next, participants answered four demographic questions.

Exit and clear survey

66%

Part 2 - Survey

Please answer the following questions.

What is your age?

This question is mandatory

What is your gender?

This question is mandatory

- Male
- Female
- Other

What is your highest level of education completed?

This question is mandatory

- Less than a High School Degree
- High School Diploma
- Vocational Training
- Attended College
- Bachelor's Degree
- Graduate Degree
- Unknown

Please choose the category that describes the total amount of income you earned in 2017. Consider all forms of income, including salaries, tips, interest and dividend payments, scholarship support, student loans, parental support, social security, alimony, and child support, and others.

This question is mandatory

- Under $5,000
- $5,000 - $10,000
- $10,001 - $15,000
- $15,001 - $25,000
- $25,001 - $35,000
- $35,001 - $50,000
- $50,001 - $65,000
- $65,001 - $80,000
- $80,001 - $100,000
- Over $100,000

Final page:

Exit and clear survey

83%

Part 2 - Survey

Thank you very much for your participation.

For your payment, please state your Worker ID here. We will not use it for any other purpose.

Note, that your Worker ID can be found on your dashboard page.

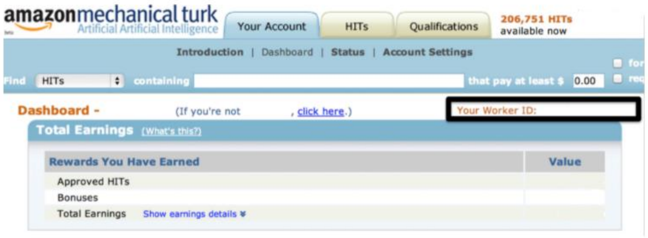

753898620721

Please copy the survey code below to paste it into the box in MTurk to receive credit for taking our survey.

Your Code: 4057685

Submit
